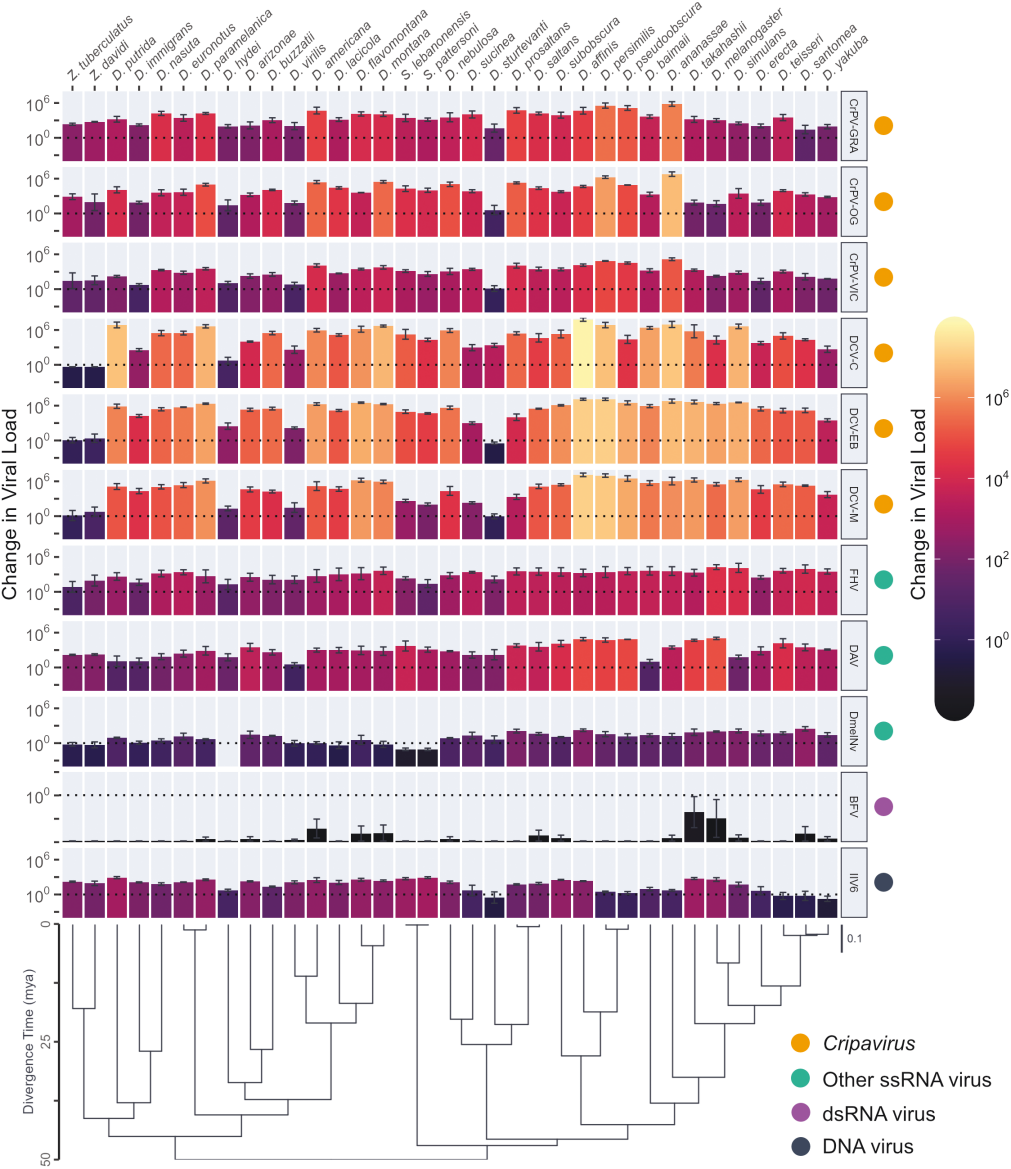# Correction to: Positive correlations in susceptibility to a diverse panel of viruses across Drosophilidae host species

**DOI:** 10.1093/evlett/qraf010

**Published:** 2025-04-11

This is a correction to:

Ryan M Imrie, Megan A Wallace, Ben Longdon, Positive correlations in susceptibility to a diverse panel of viruses across Drosophilidae host species, *Evolution Letters*, 2025, qraf002, https://doi.org/10.1093/evlett/qraf002

In the originally published version of this manuscript, the order of the data and species labels in Figure 1 were reversed with respect to the phylogeny.

To correct this error in the article, Figure 1 has been replaced with a version showing the correct orientation of the data and species labels.