# Supplementary material for: Positive correlations in susceptibility to a diverse panel of viruses across Drosophilidae host species
Source: Evol Lett. 2025 Feb 5;9(3):335–45. doi: 10.1093/evlett/qraf002 (PMC7617412; doi:10.1093/evlett/qraf002)
Supplement: qraf002_suppl_Supplementary_Material [file qraf002_suppl_supplementary_material.pdf]

Supplementary Materials

The following document contains supplementary methods, tables, and figures for Imrie et al., Positive correlations in susceptibility to a diverse panel of viruses across host species (2024).

Supplementary Methods

Phylogenetic Generalised Linear Mixed Model Structures

Three structures of phylogenetic generalised linear mixed models were used in this study: a univariate structure that included a non-phylogenetic species-specific random effect (1), which was used to estimate phylogenetic heritabilities; a univariate structure with this non-phylogenetic random effect removed, which was used to estimate repeatabilities (2); and a bivariate version of (2) was used to estimate phylogenetic correlations between pairs of viruses (3):

$$y_{hi} = \beta_1 + \mu_{p:h} + \mu_{s:h} + e_{hi} \; (1)$$

$$y_{hi} = \beta_1 + \mu_{p:h} + e_{hi} \; (2)$$

$$y_{hiv} = \beta_{1:v} + \mu_{p:hv} + e_{hiv} \; (3)$$

In these models, y_{hiv} is the change in viral load for virus v in the ith biological replicate of host species h. The fixed effect β_1 represents the intercepts for each virus isolate, the random effect μ_p represents the effects of the host phylogeny assuming a Brownian motion model of evolution, μ_s represents the non-phylogenetic species-specific random effect, and e represents the model residuals.

Within each of these models the random effects and residuals were assumed to follow a multivariate normal distribution with a centred mean of zero and a covariance structure of $V_p \otimes A$ for the phylogenetic effects, $V_s \otimes I$ for species-specific effects, and $V_e \otimes I$ residuals, where ⊗ represents the Kronecker product. A represents the host phylogenetic relatedness matrix, I an identity matrix and V represents either a 1x1 or 2x2 covariance matrix describing the between-species variances and covariances of changes in viral load for the different viruses. Specifically, the matrices V_p and V_s describe the phylogenetic and non-phylogenetic between-species variances in viral load for each virus and the covariances between them, while the residual covariance matrix V_e describes within-species variance that includes both true within-species effects and measurement errors. Since each biological replicate was tested with a single virus isolate, the covariances of V_e cannot be estimated and were set to zero.

Models were run for 13 million MCMC generations, sampled every 5,000 iterations with a burn-in of 3 million generations. Parameter expanded priors were placed on the covariance matrices, resulting in multivariate F distributions with marginal variance distributions scaled by 1,000. Inverse-gamma priors were placed on the residual variances, with a shape and scale equal to 0.002. To ensure the model outputs were robust to changes in prior distribution, models were also fitted with flat and inverse-Wishart priors, which gave qualitatively similar results.

Supplementary Tables

	Antiviral RNAi		Toll		IMD		JAK-STAT		STING		Phagocytosis	
	Controls Virus	Suppressed	Controls Virus	Suppressed	Controls Virus	Suppressed	Controls Virus	Suppressed	Controls Virus	Suppressed	Controls Virus	Suppressed
**CrPV**	[1–4]	[5]	[6]		[4,7]		[8]		[9]		[10]	
**DCV**	[1–4,11]	[1]	[6]		[4,12]		[8,13]		[14]		[15,16]	
**FHV**	[2–4,11,17]	[18]	[6]		[19]		[19]		[4]		[10]	
**DAV**	[20]				[21]				[21]			
**DmelNV**	[22]	[23]	[6,22]				[22]					
**BFV**												
**IIV6**	[4,8,24,25]	[25]		[26]		[26]	[27]		[4]		[10]	

Supplementary Table 1: Known interactions between each virus species included in this study and *D. melanogaster* antiviral immune pathways. Dark grey boxes indicate immune pathways that control or become suppressed by the virus, while white boxes indicate pathways that do not control or become suppressed by the virus. Light grey boxes indicate combinations of immune pathway and virus where no/insufficient evidence is available. Results shown have been taken from a combination of *in vitro* and *in vivo* experimental studies, references for which are provided in (Supplementary Table 1). Responses that are known to be context dependent (*e.g.,* dose or infection route dependent) are marked with an asterisk (*), and the response most relevant to the infection context of this study is displayed. References for these effects are given inside each cell.

	CrPV-OG			CrPV-GRA			CrPV-VIC			DCV-C			DCV-EB			DCV-M			FHV			DAV			IIV6			DmelNv			BFV		
D. affinis	15	15	14	10	13	15	14	13	14	8	15	12	14	14	13	14	15	13	14	14	12	14	14	12	9	15	15	15	14	15	15	11	15
D. americana	12	15	15	9	15	15	14	11	14	12	10	15	14	15	15	15	15	12	15	13	14	13	13	14	15	15	15	12	14	15	15	14	14
D. ananassae	13	14	15	14	14	14	15	15	15	15	14	15	13	13	13	12	12	14	15	14	13	11	14	15	10	12	14	14	14	12	14	15	11
D. arizonae	15	14	14	10	15	14	12	14	14	14	13	15	15	15	13	13	15	14	14	15	15	10	13	15	13	15	12		15	13	15	15	12
D. baimaii	12	14	10	13	15	14	12	11	11	9	14	14	12	12	12	12	11	14	12	15	12	8	14	14	15	15	11	15	10	12	10	10	12
D. buzzatii	14	13	14	7	15	15	14	14	10	13	13	14	14	15	15	15	12	15	15	15	14	12	15	15	12	14	14	13	15	13	15	15	15
D. erecta	13	13	15	13	14	15	14	15	15	13	13		13	13	14	14	11	10	14	13	13	13	15	13	15	11	15		13	14	13	14	15
D. euronotus	13	15	14	14	15	12	14	13	15	13	15	13	15	13	10	14	13	15	14	11	14	11	14	15	15	15	14		14	14	15	14	15
D. flavomontana	14	13	13	14	14	15	13	15	15	12	13	15	15	14	14	15	15	14	14	15	15	15	15	15	15	14	15	15	15	14	14	15	15
D. hydei	15	15	15	14	15	15	13	15	14	15	14	14	15	14	14	15	15	15	13	15	8	15	15	15	15	15	15				15	14	14
D. immigrans		11	14		15	12	15	15	15	13	14	13	12	15	13		13	13	14	8	15	15	15	11	14	10	14	14	15	14	15	14	14
D. lacicola	14	12	11	13	15	12	15	12	15	12	11	14	15	13	14	14	12	13	13	11	10	14	14	15	15	14	14	15	13	14	15	13	14
D. melanogaster	15	15	15	13	15	14	15	13	13	13	15	15	15	15	15	15	15	15	15	14	14	15	14	15	15	15	15	15	15	15	13	14	15
D. montana	15	15	15	15	15	15	15	14	15	15	15	14	15	15	14	14	15	15	15	15	15	15	15	15	15	15	12	15	13	15	15	15	15
D. nasuta	15	13	15	12	14	15	15	14	12	12	14	14	12	14	12	12	14	15	12	14	14	14	11	13	14	11	14	15	14	14	9	14	15
D. nebulosa	14	8	13	15	10	8	10	8	7	11	14	9	13	12	12	13	11	11	11	11	12	15	9	8	12	13	14	14	10	10	11	11	10
D. paramelanica	15	13	15	13	14	14	13	12	13	11	15	14	12	14	14	11	11	12	13	12	14	12	13	13	15	14	15	14	12	14	14	13	14
D. persimilis	15	14	12	15	15	15	15	12	13	15	15	15	14	14	15	14	14	14	15	15	12	14	14	12	13	14	15	14	13	14	15	14	11
D. prosaltans	15	15	13	15	13	13	15	13	15	14	15	15	15	14	15	15	15	14	15	15	15	15	14	14	15	14	13	13	15	15	15	15	11
D. pseudoobscura	15	15	15	14	15	14	14	15	13	14	15	14	15	15	14	14	14	13	15	14	15	14	15	13	14	14	15	15	15	15	15	13	15
D. putrida	15	13	14	13	15	13	13	13	9	12	14	13	15	15	12	14	11	15	15	13	13	11	11	11	13	14	14	13	11	12	12	15	11
D. saltans	15	15	14	13	15	15	15	15	14	15	12	15	15	14	13	15	14	15	14	14	15	15	15	15	15	14	15	15	15	13	14	13	14
D. santomea	14	15	11	14	15	15	14	15	14	14	11	13	13	12	15	13	13	13	13	15	14	13	13	15	14	14	13	13	13	14	12	13	15
D. simulans	15	14	15	15	14	14	15	15	15	11	15	14	15	14	14	15	14	15	14	15	15	12	15	15	14	15	14	14	14	14	14	15	15
D. sturtevanti	8	7	13	12	8	13	7	9	12	13		15	14	7	13	11	7	15	15	8	15	10	7	13	8	7	13	10	8	12	14	8	9
D. subobscura	14	13	15	14	14	15	14	14	15	12	10	13	15	15	14	14	14	14	12	15	14	14	15	15	7	14	15	10	13	15	10	14	15
D. sucinea	13	9	14	13	14	14	12	10	15	9	11	14	14	14	14	10	13	12	11	12	12	10	11	7	10	12	13	12	10	15	12	13	9
D. takahashii	15	15	15	11	15	15	13	15	15	15	13	15	15	15	15	15	13	15	15	15	15	9	15	14	13	15	15	15	15	14	12	15	15
D. teisseri	13	14	13	15	15	15	14	12	14	15	13	14	11	13	12	9	12	14	12	15	14	10	14	15	15	13	14	14	12	15	14	12	14
D. virilis	15	15	15	15	13	15	15	15	14	15	15	15	14	15	15	15	13	14	15	15	14	15	15	15	15	15	15		14	15	15	15	14
D. yakuba	10	13	12	11	13	13	12	12	11	10	11	11	10	13	12	12	9	11	13	9	14	10	10	12	13	15	11	11	7	12	11	15	13
S. lebanonensis	15	14	15	15	15	14	12	13	15	15	14		14	14	15	15	15	14	10	15	15	15	9	13	14	15	15	15	14	15	13	15	15
S. pattersoni	15	15	15	14	11	14	15	14	13	15	15	14	15	15	15	14	15	15	13	15	14	15	15	15	15	7	15	15	15	15	14	15	14
Z. davidi	15	15	15	15	14	15	15	15	12	13	15	14	15	15	10	10	15	14	15	15	15	13	15	15	13	15		15	15	15	15	15	15
Z. tuberculatus	15	14	14	15	15	14	15	14	15	15	15	15	15	15	15	14	15	14	14	15	13	14	14	14	12	13	15	15	15	15	13	14	14

Supplementary Table 2: Biological replicates (vials) and flies per vial by species and virus isolate. Black boxes indicate replicates that were excluded from analysis, all of which were removed due to melt-curve contaminants.

Isolate	NCBI Accession
CrPV-BEE	PQ246907
CrPV-GRA	PQ246908
CrPV-KKH	PQ246909
CrPV-KTA	PQ246910
CrPV-NEU	PQ246911
CrPV-OG	PQ246912
CrPV-VIC	PQ246913
CrPV-WHP	PQ246914
DCV-C	MK645242.1
DCV-CYG	MK645238.1
DCV-EB	MK645239.1
DCV-G	MK645241.1
DCV-M	MK645243.1
DCV-O	MK645244.1
DCV-T	MK645245.1
DCV-Z	MK645240.1

Supplementary Table 3: DCV and CrPV isolate NCBI Genome Accessions

Virus	Forward	Reverse
CrPV	GGAGAACCGATTTCGTATGA	GTTGGTGGAATGTCTTCTCT
DCV	GACACTGCCTTTGATTAG	CCCTCTGGGAACTAAATG
FHV	TTATTATGTCACCGAGCCTG	CTTCGGGTTAAAGGTGTGTA
DAV	TATCTTACCAAAGGCAACCC	CAAACTCAATCACCCATTCG
DmelNv	TGGTTTGTATGCGTGGGTGA	GCTCGAGACATTCTGTCGGT
BFV	ATGTTGACTCGACGTACTAC	GCGTATTTCAAAGCATGACA
IIV6	GAACACAACAAACCGTTTCC	GGTGCAGATGGTGTAACAAT

Supplementary Table 4: Viral q-PCR primers

Direction	Name	Sequence
Forward	F-a	TGCCAAGTTGTCGCACAAATGG
	F-b	TGCTAAGTTGTCGCACAAATGG
	F-c	TGCCAAGCTGTCGCACAAATGG
	F-d	TGCTAAGCTGTCGCACAAATGG
	F-e	TGCGAAGTTGTCGCACAAATGG
	F-f	TGCGAAGCTGTCGCACAAATGG
Reverse (cDNA)	R-a	TGCGCTTGTTGGAACCGTAAC
	R-b	TGCGCTTGTTGGATCCGTAAC
	R-c	TGCGCTTGTTGGAACCATAAC
	R-d	TGCGCTTGTTGGAGCCGTAAC
	R-e	TGCGCTTGTTAGAACCGTAAC
	R-f	TACGCTTGTTGGAACCGTAAC
	R-g	TGCGCTTGTTGGAACCGTAGC
	R-h	TGCGCTTGTTCGATCCGTAAC
	R-i	TGCGCTTGTTGGAGCCATAAC
	R-j	TGCGCTTGTTTGATCCGTAAC
	R-k	TGCGCTTGTTTGAACCATAAC
	R-l	TACGCTTGTTGGAACCATAAC
	R-m	TACGCTTGTTGGAGCCGTAAC
	R-n	TGCGCTGGTTGGAACCATAAC
	R-o	TGAGCTTGTTCGATCCGTAAC
	R-p	TACGCTTGTTGGAGCCATAAC
	R-q	TGAGCTTGTTTGATCCGTAAC
	R-r	TAAGCTTGTTGGATCCGTAGC
	R-s	TCAGCTTGTTGGATCCATAGC
Reverse (gDNA)	R-gDNA	GGYTTRCGCCATTTGTGC

Supplementary Table 5: RPL32 q-PCR primers

Species	Forward	Reverse (cDNA)	Reverse (gDNA)
D. affinis	F-a	R-i	R-gDNA
D. americana	F-c	R-a	R-gDNA
D. ananassae	F-f	R-a	R-gDNA
D. arizonae	F-a	R-a	R-gDNA
D. baimaii	F-a	R-r	R-gDNA
D. buzzati	F-a	R-e	R-gDNA
D. erecta	F-d	R-h	R-gDNA
D. euronotus	F-a	R-g	R-gDNA
D. flavomontana	F-c	R-a	R-gDNA
D. hydei	F-a	R-a	R-gDNA
D. immigrans	F-b	R-p	R-gDNA
D. lacicola	F-c	R-a	R-gDNA
D. melanogaster	F-d	R-h	R-gDNA
D. montana	F-c	R-a	R-gDNA
D. nasuta	F-b	R-f	R-gDNA
D. nebulosa	F-b	R-c	R-gDNA
D. paramelanica	F-a	R-g	R-gDNA
D. persimilis	F-a	R-b	R-gDNA
D. prosaltans	F-a	R-n	R-gDNA
D. pseudoobscura	F-a	R-m	R-gDNA
D. putridia	F-d	R-q	R-gDNA
D. saltans	F-a	R-n	R-gDNA
D. santomea	F-a	R-n	R-gDNA
D. simulans	F-d	R-h	R-gDNA
D. sturtevanti	F-a	R-l	R-gDNA
D. subobscura	F-a	R-i	R-gDNA
D. sucinea	F-b	R-k	R-gDNA
D. takahashii	F-d	R-o	R-gDNA
D. teissieri	F-d	R-h	R-gDNA
D. virilis	F-c	R-a	R-gDNA
D. yakuba	F-d	R-h	R-gDNA
S. lebanonensis	F-d	R-h	R-gDNA
S. pattersoni	F-a	R-m	R-gDNA
Z. davidi	F-a	R-c	R-gDNA
Z. tuberculatus	F-a	R-c	R-gDNA

Supplementary Table 6: RPL32 Primer Combinations

Cycle step	Temp.	Time/rate	Cycle No.
Initial denaturation	95˚C	2 min	1
Denaturation	95˚C	5 sec	40
Annealing/Extension	60˚C	15 sec	
Melt curve	60˚C - 95˚C	0.1˚C/s	1

Supplementary Table 7: q(RT)-PCR Cycle Conditions

Virus	Repeatability	Phylogenetic Heritability
CrPV-OG	**0.84 (0.74, 0.93)**	0.47 (0.00, 0.97)
CrPV-GRA	**0.69 (0.51, 0.86)**	**0.73 (0.19, 1.00)**
CrPV-VIC	**0.82 (0.71, 0.92)**	**0.75 (0.13, 1.00)**
DCV-C	**0.90 (0.82, 0.96)**	**0.72 (0.23, 1.00)**
DCV-EB	**0.96 (0.93, 0.98)**	**0.86 (0.52, 1.00)**
DCV-M	**0.87 (0.79, 0.94)**	**0.87 (0.59, 1.00)**
FHV	**0.22 (0.04, 0.42)**	**0.85 (0.39, 1.00)**
DAV	**0.62 (0.4, 0.82)**	**0.79 (0.35, 1.00)**
DmelNV	**0.57 (0.38, 0.75)**	**0.94 (0.77, 1.00)**
BFV	0.17 (0, 0.41)	0.54 (0.01, 1.00)
IIV6	**0.77 (0.64, 0.88)**	**0.95 (0.78, 1.00)**

Supplementary Table 8: Estimates of repeatability and phylogenetic heritability in viral load for each virus. All models were fitted on $\log_{10}$ transformed fold-changes in viral load. Values for phylogenetic heritability are taken from a model containing a non-phylogenetic species-specific random effect, while values for repeatability are taken from a model with this effect removed. Estimates that are credibly non-zero are shown in bold.

Virus	Wing length
CrPV-OG	0.18 (-2.18, 2.38)
CrPV-GRA	-0.18 (-1.86, 1.38)
CrPV-VIC	-0.37 (-1.93, 1.44)
DCV-C	0.13 (-2.51, 3.01)
DCV-EB	-0.84 (-3.31, 1.56)
DCV-M	-1.12 (-3.74, 1.12)
FHV	-0.44 (-1.65, 0.91)
DAV	-0.08 (-1.73, 1.49)
DmelNV	-1.16 (-2.20, 0.06)
BFV	-0.03 (-0.79, 0.79)
IIV6	0.27 (-1.00, 1.76)

Supplementary Table 9: Estimates of the effect of wing length (a proxy of body size) on viral load for each virus.

Group	Correlation Coefficient (R)
Within Species	0.93 (0.82, 0.99)
Within Family	0.73 (0.49, 0.93)
Across Family	0.33 (-0.18, 0.79)

Supplementary Table 10: Estimates of the strength of correlations between viruses at different evolutionary scales. Posterior distributions of the correlation coefficients for each virus pair were combined to create overall distributions for correlations within virus species, within virus family, and across virus family.

Group	Correlation Coefficient (R)
Within Species	0.93 (0.82, 0.99)
Within Family	0.73 (0.49, 0.93)
Across Family	0.27 (-0.11, 0.63)

Supplementary Table 11: Estimates of the strength of correlations between viruses at different evolutionary scales with BFV included.

Supplementary Figures

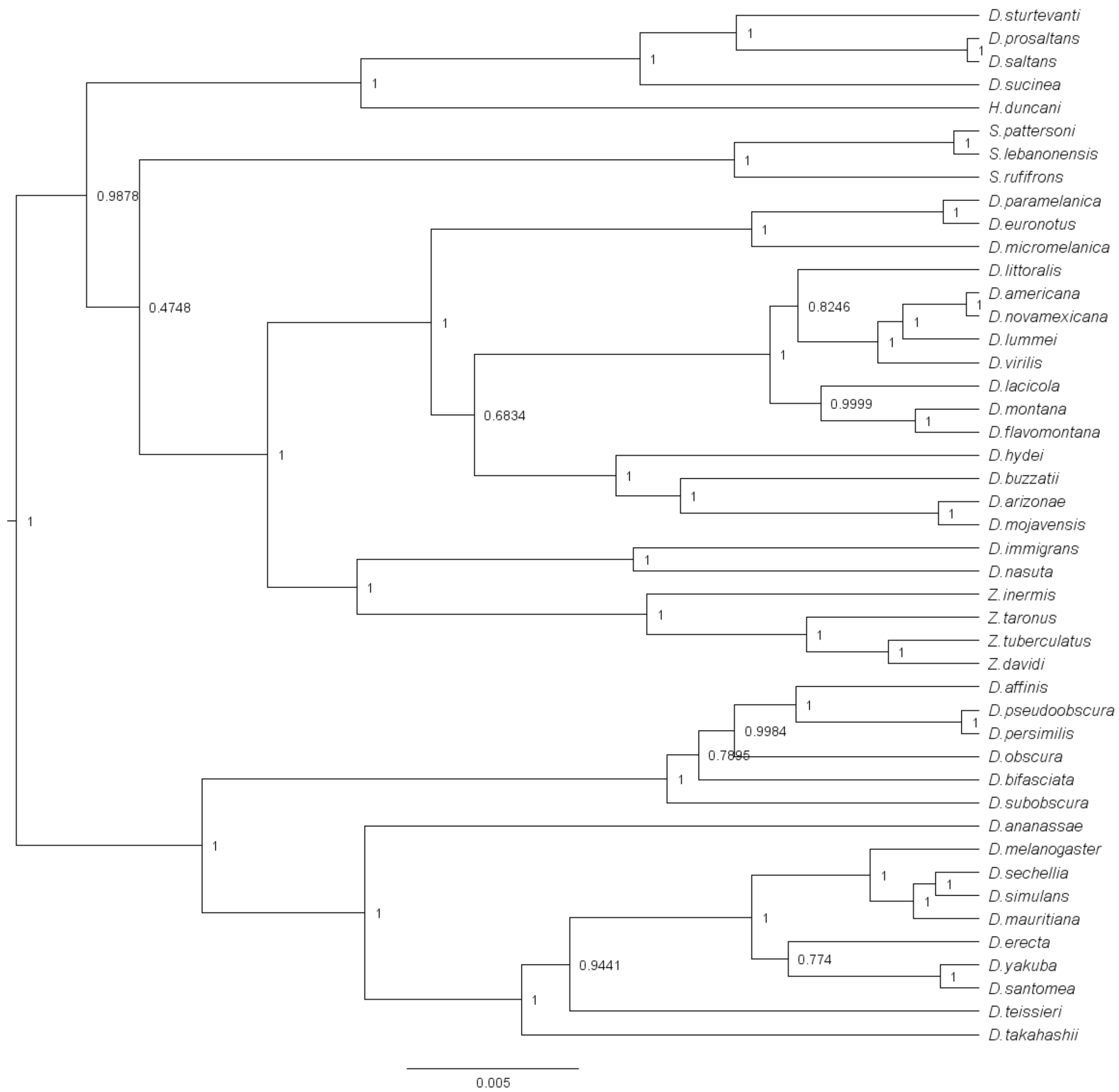

Supplementary Figure 1: Phylogeny of *Drosophilidae* host species. Evolutionary relationships are presented as a midpoint rooted, maximum clade credibility tree. Node labels are the posterior probabilities of each clade, and the scale bar represents nucleotide substitutions per site.

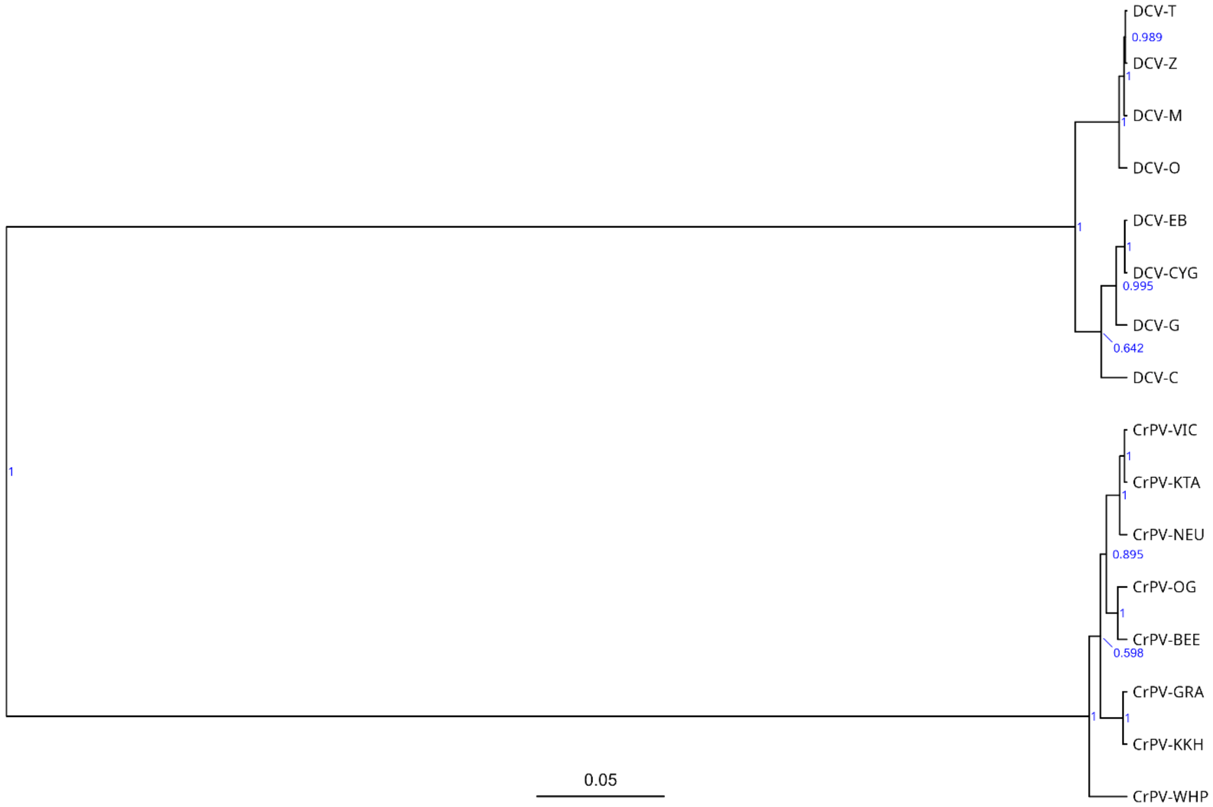

Supplementary Figure 2: Phylogeny of DCV and CrPV isolates. Evolutionary relationships are presented as a midpoint rooted, maximum clade credibility tree. Node labels (blue) are the posterior probabilities of each clade, and the scale bar represents nucleotide substitutions per site.

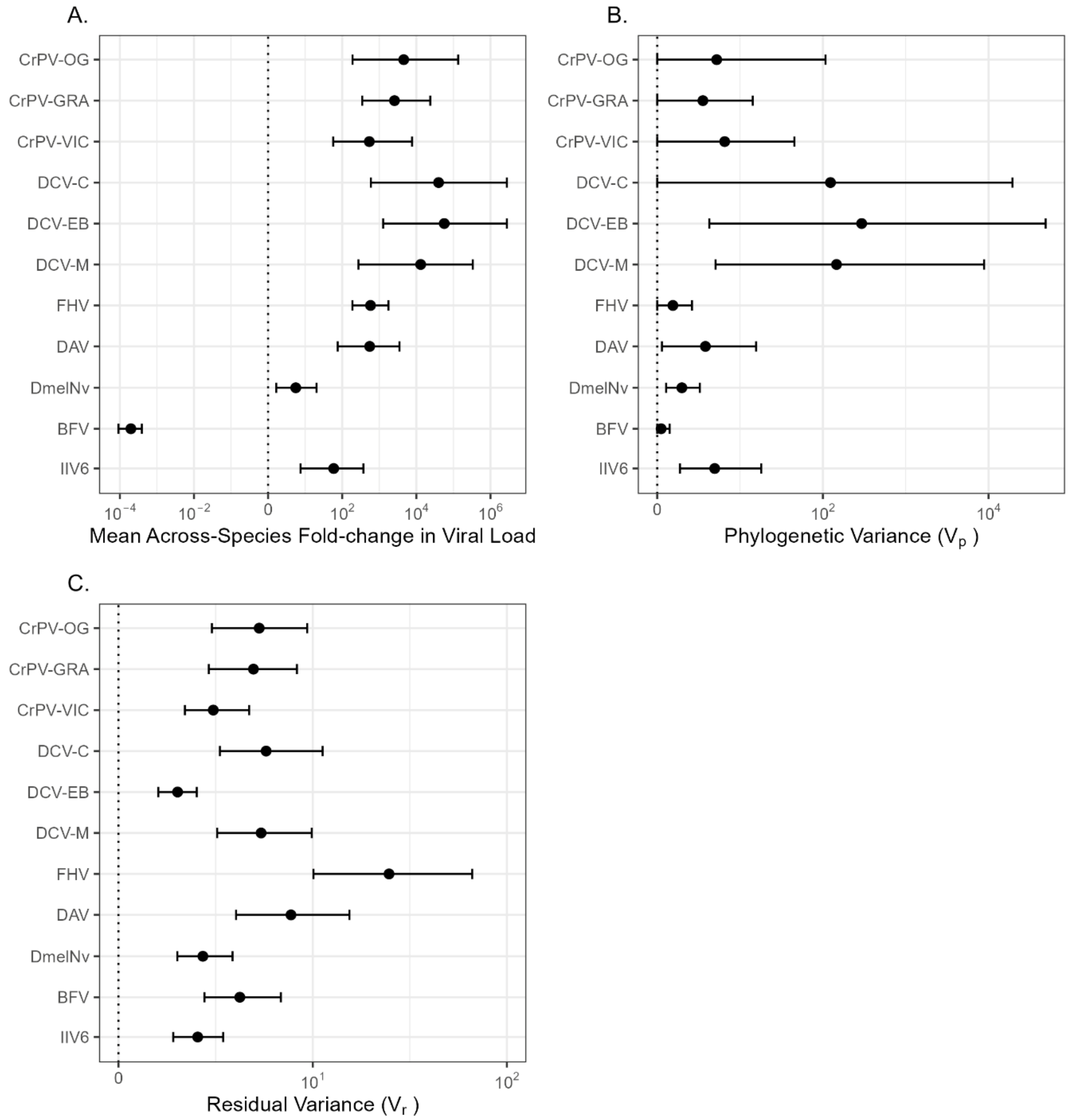

Supplementary Figure 3: Estimates of the mean across species viral loads and variances for each virus. Values for the mean across species fold-change in viral load (A), phylogenetic variance (B), and residual variance (C) were taken from univariate models with the non-phylogenetic species-specific random effect removed.

References

1. Rij RP van, Saleh M-C, Berry B, Foo C, Houk A, Antoniewski C, et al. The RNA silencing endonuclease Argonaute 2 mediates specific antiviral immunity in Drosophila melanogaster. Genes Dev. 2006;20: 2985–2995. doi:10.1101/gad.1482006

2. Wang X-H, Aliyari R, Li W-X, Li H-W, Kim K, Carthew R, et al. RNA Interference Directs Innate Immunity Against Viruses in Adult Drosophila. Science. 2006;312: 452–454. doi:10.1126/science.1125694

3. Mondotte JA, Gausson V, Frangeul L, Blanc H, Lambrechts L, Saleh M-C. Immune priming and clearance of orally acquired RNA viruses in Drosophila. Nat Microbiol. 2018;3: 1394–1403. doi:10.1038/s41564-018-0265-9

4. Goto A, Okado K, Martins N, Cai H, Barbier V, Lamiable O, et al. The Kinase IKKβ Regulates a STING- and NF-κB-Dependent Antiviral Response Pathway in Drosophila. Immunity. 2018;49: 225-234.e4. doi:10.1016/j.immuni.2018.07.013

5. Nayak A, Berry B, Tassetto M, Kunitomi M, Acevedo A, Deng C, et al. Cricket paralysis virus antagonizes Argonaute 2 to modulate antiviral defense in Drosophila. Nat Struct Mol Biol. 2010;17: 547–554. doi:10.1038/nsmb.1810

6. Ferreira ÁG, Naylor H, Esteves SS, Pais IS, Martins NE, Teixeira L. The Toll-Dorsal Pathway Is Required for Resistance to Viral Oral Infection in Drosophila. PLoS Pathog. 2014;10: e1004507. doi:10.1371/journal.ppat.1004507

7. Costa A, Jan E, Sarnow P, Schneider D. The Imd Pathway Is Involved in Antiviral Immune Responses in Drosophila. PLoS ONE. 2009;4: e7436. doi:10.1371/journal.pone.0007436

8. Kemp C, Mueller S, Goto A, Barbier V, Paro S, Bonnay F, et al. Broad RNA Interference–Mediated Antiviral Immunity and Virus-Specific Inducible Responses in Drosophila. J Immunol. 2013;190: 650–658. doi:10.4049/jimmunol.1102486

9. Hédelin L, Thiébaut A, Huang J, Li X, Lemoine A, Haas G, et al. Investigating the Evolution of Drosophila STING-Dependent Antiviral Innate Immunity by Multispecies Comparison of 2′3′-cGAMP Responses. Mol Biol Evol. 2024;41: msae032. doi:10.1093/molbev/msae032

10. Lamiable O, Arnold J, Faria IJ da S de, Olmo RP, Bergami F, Meignin C, et al. Analysis of the Contribution of Hemocytes and Autophagy to Drosophila Antiviral Immunity. J Virol. 2016;90: 5415–5426. doi:10.1128/jvi.00238-16

11. Sabin LR, Zhou R, Gruber JJ, Lukinova N, Bambina S, Berman A, et al. Ars2 Regulates Both miRNA- and siRNA- Dependent Silencing and Suppresses RNA Virus Infection in Drosophila. Cell. 2009;138: 340–351. doi:10.1016/j.cell.2009.04.045

12. Sansone CL, Cohen J, Yasunaga A, Xu J, Osborn G, Subramanian H, et al. Microbiota-Dependent Priming of Antiviral Intestinal Immunity in Drosophila. Cell Host Microbe. 2015;18: 571–581. doi:10.1016/j.chom.2015.10.010

13. Dostert C, Jouanguy E, Irving P, Troxler L, Galiana-Arnoux D, Hetru C, et al. The Jak-STAT signaling pathway is required but not sufficient for the antiviral response of drosophila. Nat Immunol. 2005;6: 946–953. doi:10.1038/ni1237

14. Cai H, Li L, Slavik KM, Huang J, Yin T, Ai X, et al. The virus-induced cyclic dinucleotide 2′3′-c-di-GMP mediates STING-dependent antiviral immunity in Drosophila. Immunity. 2023;56: 1991-2005.e9. doi:10.1016/j.immuni.2023.08.006

15. Nainu F, Tanaka Y, Shiratsuchi A, Nakanishi Y. Protection of Insects against Viral Infection by Apoptosis-Dependent Phagocytosis. J Immunol. 2015;195: 5696–5706. doi:10.4049/jimmunol.1500613

16. Ye T, Zhang X. Involvement of Ran in the regulation of phagocytosis against virus infection in S2 cells. Dev Comp Immunol. 2013;41: 491–497. doi:10.1016/j.dci.2013.07.015

17. Galiana-Arnoux D, Dostert C, Schneemann A, Hoffmann JA, Imler J-L. Essential function in vivo for Dicer-2 in host defense against RNA viruses in drosophila. Nat Immunol. 2006;7: 590–597. doi:10.1038/ni1335

18. Singh G, Popli S, Hari Y, Malhotra P, Mukherjee S, Bhatnagar RK. Suppression of RNA silencing by Flock house virus B2 protein is mediated through its interaction with the PAZ domain of Dicer. FASEB J. 2009;23: 1845–1857. doi:10.1096/fj.08-125120

19. Shen R, Zheng K, Zhou Y, Chi X, Pan H, Wu C, et al. A dRASSF-STRIPAK-Imd-JAK/STAT axis controls antiviral immune response in Drosophila. Cell Rep. 2022;40: 111143. doi:10.1016/j.celrep.2022.111143

20. Brosh O, Fabian DK, Cogni R, Tolosana I, Day JP, Olivieri F, et al. A novel transposable element-mediated mechanism causes antiviral resistance in Drosophila through truncating the Veneno protein. Proc Natl Acad Sci. 2022;119: e2122026119. doi:10.1073/pnas.2122026119

21. Nigg JC, Castelló-Sanjuán M, Blanc H, Frangeul L, Mongelli V, Godron X, et al. Viral infection disrupts intestinal homeostasis via Sting-dependent NF-κB signaling in Drosophila. Curr Biol. 2024;34: 2785-2800.e7. doi:10.1016/j.cub.2024.05.009

22. Habayeb MS, Cantera R, Casanova G, Ekström J-O, Albright S, Hultmark D. The Drosophila Nora virus is an enteric virus, transmitted via feces. J Invertebr Pathol. 2009;101: 29–33. doi:10.1016/j.jip.2009.02.003

23. Mierlo JT van, Overheul GJ, Obadia B, Cleef KWR van, Webster CL, Saleh M-C, et al. Novel Drosophila Viruses Encode Host-Specific Suppressors of RNAi. PLoS Pathog. 2014;10: e1004256. doi:10.1371/journal.ppat.1004256

24. Bronkhorst AW, Cleef KWR van, Vodovar N, İnce İA, Blanc H, Vlak JM, et al. The DNA virus Invertebrate iridescent virus 6 is a target of the Drosophila RNAi machinery. Proc Natl Acad Sci. 2012;109: E3604–E3613. doi:10.1073/pnas.1207213109

25. Bronkhorst AW, Cleef KWR van, Venselaar H, Rij RP van. A dsRNA-binding protein of a complex invertebrate DNA virus suppresses the Drosophila RNAi response. Nucleic Acids Res. 2014;42: 12237–12248. doi:10.1093/nar/gku910

26. West C, Rus F, Chen Y, Kleino A, Gangloff M, Gammon DB, et al. IIV-6 Inhibits NF-κB Responses in Drosophila. Viruses. 2019;11: 409. doi:10.3390/v11050409

27. West C, Silverman N. p38b and JAK-STAT signaling protect against Invertebrate iridescent virus 6 infection in Drosophila. PLoS Pathog. 2018;14: e1007020. doi:10.1371/journal.ppat.1007020
